# Diagnostic controversies in recurrent painful ophthalmoplegic neuropathy: single case report with a systematic review

**DOI:** 10.1186/s13052-022-01274-x

**Published:** 2022-06-03

**Authors:** Raffaele Falsaperla, Santiago Presti, Manuela Lo Bianco, Stefano Catanzaro, Silvia Marino, Martino Ruggieri

**Affiliations:** 1 Unit of Pediatrics and Pediatric Emergency, University Hospital “Policlinico Rodolico-San Marco”, Catania, Italy; 2 Unit of Neonatal Intensive Care and Neonatology, University Hospital “Policlinico Rodolico-San Marco”, Catania, Italy; 3 Department of Clinical and Experimental Medicine, Pediatrics Postgraduate Residency Program, Section of Pediatrics and Child Neuropsychiatry, University of Catania, Catania, Italy; 4 Unit of Pediatrics Hospital “Umberto I”, Siracusa, Italy; 5 Department of Clinical and Experimental Medicine, Unit of Rare Diseases of the Nervous System in Childhood, Section of Paediatrics and Child Neuropsychiatry, University of Catania, Catania, Italy

**Keywords:** Recurrent painful ophthalmoplegic neuropathy, Ophthalmoplegic migraine, Schwannoma, Case report, Ophthalmoplegia, Headache

## Abstract

**Background:**

Ophthalmoplegic migraine, renamed "Recurrent Painful Ophthalmoplegic Neuropathy" (RPON) in 2013 by the *International Headache Society* is a rare neurologic disorder characterized by recurrent attacks of ophthalmoplegia associated to ipsilateral headache. The etiology is still unknown. Typical magnetic resonance imaging findings show a focal nerve thickening and contrast enhancement. In the majority of cases, there is a full recovery within days or weeks. There is no evidence supporting a specific treatment. The review defines the characteristics of the recurrent painful ophthalmoplegic neuropathy in patients within 2 years of age underlying the importance of the role of magnetic resonance imaging even in presence of the first attack. Thus, an emblematic case report is presented.

**Case presentation:**

The authors present a case of third cranial nerve paresis in a 17-month-old male child, presenting a neuroradiological pattern highly suggestive of schwannoma, aneurism or recurrent painful ophthalmoplegic neuropathy. Thus, a review of the literature with the pediatric casuistry of recurrent painful ophthalmoplegic neuropathy occurred within 2 years of age focusing on diagnostic considerations is presented. The authors highlight the importance to consider recurrent painful ophthalmoplegic neuropathy in presence of magnetic resonance imaging findings and clinical symptoms referable to aneurysm or schwannoma. Thus, the review defines the characteristics and the neuroradiological findings at the first RPON attack occurred under 2 years of age.

**Conclusion:**

Although two attacks are necessary, the review strongly suggests to consider recurrent painful ophthalmoplegic neuropathy even at the first attack, in presence of described characteristics and the aforementioned magnetic resonance imaging findings.

## Background

Ophthalmoplegic migraine (OM), renamed “Recurrent Painful Ophthalmoplegic Neuropathy (RPON) in 2013 by the *International Headache Society *[1] is a rare neurologic disorder characterized by recurrent attacks of ophthalmoplegia associated to ipsilateral headache, that can be migranous or not. In population, incidence is estimated as 0.7/million every year. Laboratory tests such as erythrocythe sedimentation rate, rheumathoid factor, antinuclear antibody, Venereal Disease Research Laboratory (VDRL) and cerebrospinal fluid are normally negative [2]. In 80% of cases it involves the third cranial nerve [3]. Typical Magnetic Resonance Imaging (MRI) findings show a focal nerve thickening and contrast enhancement. The etiology of this rare syndrome is still unknown [4]. In the majority of cases, there is a full recovery within days or weeks, but less frequently, patients have persistent neurologic deficits [5]. According to the *International Classification of Headache Disorders (ICHD)*, it is possible to diagnose RPON/OM with at least 2 attacks of a migraine-like headache associated with paresis of the ocular cranial nerves that occurs within 4 days from the beginning of symptoms. Possible ocular findings are ophthalmoparesis, ptosis, or mydriasis. In order to perform the diagnosis, causes as tumors, infections, and thrombosis must have been excluded [4]. According to the aforementioned classification, diagnosing this disorder during the first attack is not possible, even if the MRI findings are highly suggestive of RPON. Specifically, thickening of the interested cranial nerve, with a reduced post-contrast enhancement isa typical pattern. The role of the imaging is challenging because frequently MRI is negative even in case of confirmed RPON.

It is worthy to mention that ocular nerve palsies are rare in childhood [6]. Specifically, the third cranial nerve is the less affected in children [7]. RPON is one of the rarest causes of third cranial nerve palsy [8]. Herein, we report a case of third cranial nerve paresis in a 17-month-old male child, presenting a neuroradiological pattern highly suggestive of schwannoma, aneurism or RPON. Even if the MRI at the first attack was highly suggestive of RPON, the diagnosis according to the ICHD was not possible. Thus, as shown in Tables 1 and 2*,* we systematically reviewed the pediatric cases of RPON/OM occurred within 2 years of age comparing them with our case.

**Table 1:** Reports from 1996 to 2007

**Main features**	Østergaard [2]	Østergaard [9]	Ramelli [10]	Lance [11]	Weiss [12]	Carlow [13]	Carlow [13]
Age at onset	18 mo	7 mo	20 mo	9 mo	24 mo	18 mo	18 mo
Current age	8 yr	19 yr	8 yr	16 yr	7 yr	-	-
Sex	F	F	F	F	M	F	F
CN involved (side)	III (L)	III(L)	III (R)	III (L)	III (L)	-	-
Headache (side)	Yes (starting with the 2nd episode; bilateral or left sided)	Yes (starting with her 5th episode; left-sided and eye pain)	Yes (starting with the 2nd episode at the age of 6 yr and 8 mo after a fall backwards – no apparent headache at 1st episode)	Yes (behind the left eye, described as sharp and fluctuating in intensity)	Yes (L; supraorbital)	Yes	Yes
Associated symptoms	No	Yes	No	Yes	No	-	-
Photophobia	-	-	-	Yes	-	-	-
Phonophobia	-	-	-	Yes	-	-	-
Nausea	-	-	-	Yes	-	-	-
Vomiting	-	Yes	-	Yes (sometimes)	-	-	-
Irritability	-	Yes	-	-	-	-	-
Other findings	Signs of varicella infection at 2nd episode Dizziness at 4th episode	Drowsiness	-	Attacks of screaming	-	-	-
Ocular symptoms/signs	Yes	Yes	Yes	Yes	Yes	-	-
Diplopia	NS	NS	Yes	NS	Yes	-	-
Ophthalmoplegia	Yes (not always present)	Yes	Yes	Yes	Yes	-	-
Palpebral ptosis	Yes	Yes	Yes	Yes	Yes	-	-
Pupillary dilation	Yes (poorly reactive pupil to light)	Yes	Yes (poorly reactive to light)	Yes (reactive to light with progression to unresponsive pupil)	Yes (sluggish response to light)	No	Yes
MRI findings in the acute phase	MRI perfomerd 12 days later 2nd episode onset (ptosis partially resolved)	Not performed	Yes – At second episode (not performed at the 1st episode)	Yes	Yes – performed after 2 weeks of onset (several foci of white matter hyperintensity measuring 3 mm or less identified in the dorsal periventricular region)	Yes	Yes
Nerve thickening	Yes (from the brainstem through the prepontin cistern to the carvernous sinus)	-	Yes	No	No	Yes	Yes
Post-contrast enhancement	Yes	-	Yes	Yes—at the point of exit of the nerve from the midbrain, continuing along the line of the nerve	No	Yes	Yes
Altered CSF if lumbar puncture performed	No (2nd episode)	No	No	NS	-	-	-
Headache duration	3–4 days	NS	NS	NS	NS	-	-
Ophthalmoplegia duration	NS	NS	NS	NS	NS	-	-
Interval between headache onset and ophthalmoplegia	3–4 days	1 day	4 days (second episode)	NS – 3–4 days between headache and palpebral ptosis	NS	-	-
Time to resolution of Symptoms/Signs	6–8 weeks	6 mo (1st episode)	Within 2 weeks (first episode)	From few days (2–11) to 2 months	NS	-	-
Therapy in the acute phase	Prednisone (2 mg/kg/day) for about 10 days with apparent response	NS	NS	NS	-	-	-
Follow-up	Yes ( refered migraine attacks without ophthalmoplegia)	Yes (permanent partial III CN palsy)	-	Yes (with apparent decreased number of episodes)	NS	-	-
Prophylactic therapy	-	Propranolol Metoclopramide Diazepam Acetaminophen	-	Cafergot; Aspirin; Amitriptyline; Pizotifen; Flunarizine ( 10 mg/day with apparent response)	NS	-	-
Control MRI	Performed after 3 months of the 3rd episode onset (persistent enlargement of III CN but to a lesser degree)	MRIs at 14, 15, 16-years-of-age showing persistent enlargement (from the brainstem through the prepontin cistern to the carvernous sinus)	NS	A repeat MRI scan showed enhancement of the oculomotor nerve still present but less intense; unenhanced MRIs of the brain at the ages of 12 and 14 years were normal	-	-	-
Number of acute episodes	NS ( about four episodes)	NS	NS	NS – About 38 episodes	-	-	-
Interval between episodes	Range 15 mo -3 yr	6–9 mo	NS	NS	-	-	-
Comorbidity	Migraine	-	Migraine without aura	-	Migraine	-	-
Family history of migraine	No	No	Yes (on the maternal side)	Yes (on the maternal side)	No	Yes	No

**Table 2:** Reports from 2007 to 2015

Main features	McMillan [5]	Bharucha [14]	Vecino López [15]	Vieira [16]	Riadh [17]	Ghosh [18]
Age at onset	12 mo	18 mo	Before 6 mo	9 mo	9 mo	18 mo
Current age	6 yr	16 yr	3 yr and 11 mo	7 yr	3 yr	NS
Sex	M	F	F	M	F	M
CN involved (side)	III (L)	III (R)	III(R)	III(R)	III(L)	III(R)
Headache (side)	Yes (starting with his 4th episode)	Yes (R)	-	Yes(R, frontotemporal and orbital pain)	Yes(L)	Yes(starting with 2nd episode)
Associated symptoms	No	No	-	Yes	Yes	No
Photophobia	-	-	-	Yes	-	-
Phonophobia	-	-	-	Yes	-	-
Nausea	-	-	-	Yes	Yes	-
Vomiting	-	-	-	Yes(occasional-ly during the first days of a episode)	Yes	-
Irritability	-	-	-	-	-	-
Other findings	-	-	-	-	Yes (abdominal pain)	-
Ocular symptoms/signs	Yes	Yes	Yes	Yes	Yes	Yes
Diplopia	-	Yes	NS	NS	-	Yes (starting with 2nd episode)
Ophthalmoplegia	Yes	Yes	Yes	Yes	Yes	No
Palpebral ptosis	Yes	Yes	Yes	Yes	Yes	Yes
Pupillary dilation	No—During his fourth episode, at 29-months-of-age, the authors describe a left sluggish pupil response	Yes (not reactive to light)	Yes (sluggish pupil response)	Yes	Yes (mildly dilated, reactive to light)	No
MRI findings in the acute phase	Yes	Yes (during last episode on the day of onset of symptoms; all previous MRI exams had yielded normal findings	Yes	Yes (infundibular dilatation of a perforating branch of the posterior cerebral artery emerging just above the superior cerebellar artery, adjacent to the affected nerve)	No	Yes
Nerve thickening	Yes – at the forth episode (29 mo of age; cisternal part of nerve root)	Yes (at nerve root origin)	Yes (cisternal part)	No	-	Yes [cisternal part – performed at 18 mo(first episode)]
Post-contrast enhancement	Yes – during first episode (12 mo of age; at the site of exit of nerve root) and forth episode (29 mo of age; cisternal part of nerve root)	Yes (at nerve root origin)	No	No	-	No
Altered CSF if lumbar puncture performed	No	No (during last episode)	NS	NS	NS	No
Headache duration	2–3 days (4th episode)	NS	-	3–7 days	NS	6–7 days (before development of ptosis
Ophthalmoplegia duration	From 2–3 days (1st episode) to 2–3 weeks (4th episode)	NS	3 mo	2–5 days (initially) 1–4 weeks	NS	-
Interval between headache onset and ophthalmoplegia	2–3 days (4th episode)	Within 6 h of onset	NS	At onset of pain	NS	-
Time to resolution of Symptoms/Signs	From 2–3 days (1st episode) to 2–3 weeks (4th episode)- The authors describe periodic recurrence with each episode taking longer to recupera-te	Within 1 week of symptom onset (last episode)	3 mo ( the authors report the use of botulinum toxin for squint)	1–4 weeks	NS	3 weeks (1st episode)
Therapy in the acute phase	Prednisone(2 mg/kg for 10 days) with tapering over the following week and apparent response	Methylprednisolone iv 25 mg/Kg for 5 days (at last episode, started immediately on the first day of onset)	Oral corticosteroids	Oral prednisone (1 mg/kg/day) with apparent response This treatment was used twice and the pain subsided much earlier (within 24–48 h)	3 pulses of methyl-prednisolone followed by an oral steroid therapy (1 mg/kg/day) for 1 week with gradual tapering over 6 weeks	Methylprednisolone iv 30 mg/Kg for 3 days (1st episode); Immunoglobulin iv 2 g/kg for 2 days (2nd episode)
Follow-up	At the age of 6 years, periodic recurrence of complete left III CN paresis, with each episode taking longer to recuperate – episodes of migraine without aura—permanent neurological damage with relative mydriasis (reactive to light)		Yes	Yes ( not fully recovering from ophthalmople-gia)	No episodes	Normal neurologic examination
Prophylactic therapy	Pizotifen (beneficial for migraine,not for ophthalmople-gia)		-	Flunarizine (decreased number of episodes)	-	-
Control MRI	MRI at 15 mo of age with normal findings	Yes (at 3 and 7 months after the onset of symptoms with demonstrated reversal of abnormalities)	MRI after four mo of onset (reduced III CN enlargement)	NS	-	-
Number of acute episodes	NS ( the authors describes surely foru episodes at 12, 17, 23 and 29-months-of-age)	8	NS	NS	4 (9 mo, 1y, 2y, 3y)	3 ( 18mo, 3y, 5y)
Interval between episodes	NS	-	NS	From weeks to months	Range 3–12 mo	Range 16–24 mo
Comorbidity	No	No	-	No	-	-
Family history of migraine	No	-	-	Yes (on the maternal side)	Yes	Yes (on the maternal side)

## Case presentation

**Fig. 1:**
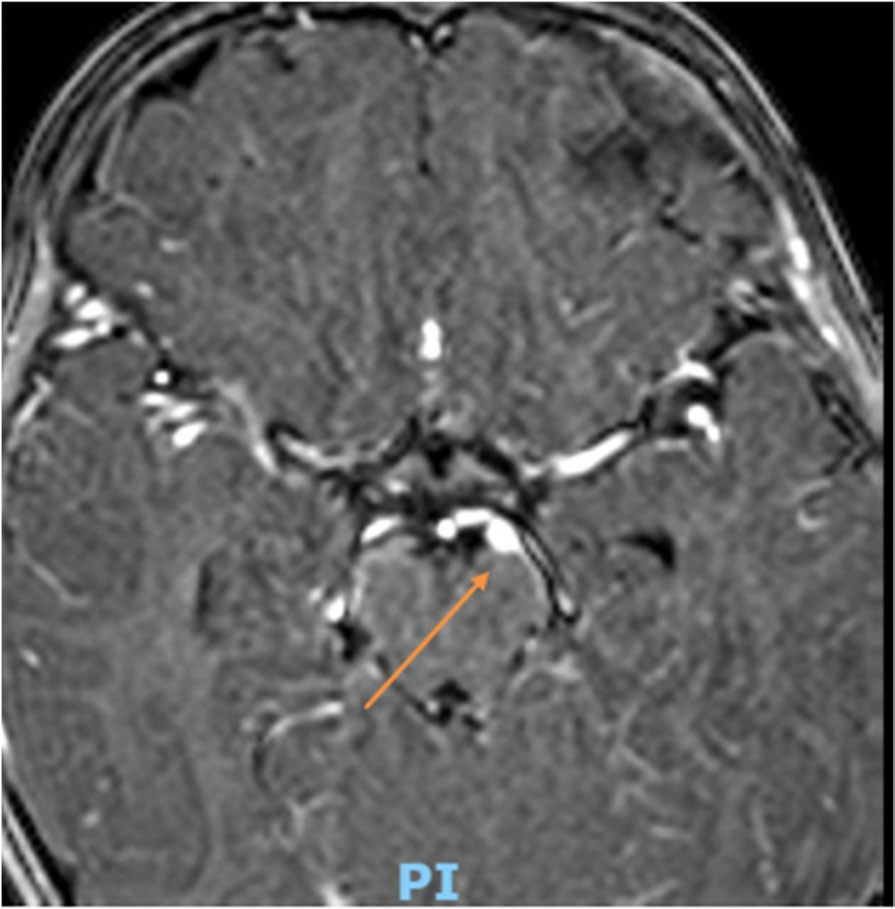
First MRI wrongly suggestive for an aneurism along the medial side of the left cerebral peduncle

**Fig. 2:**
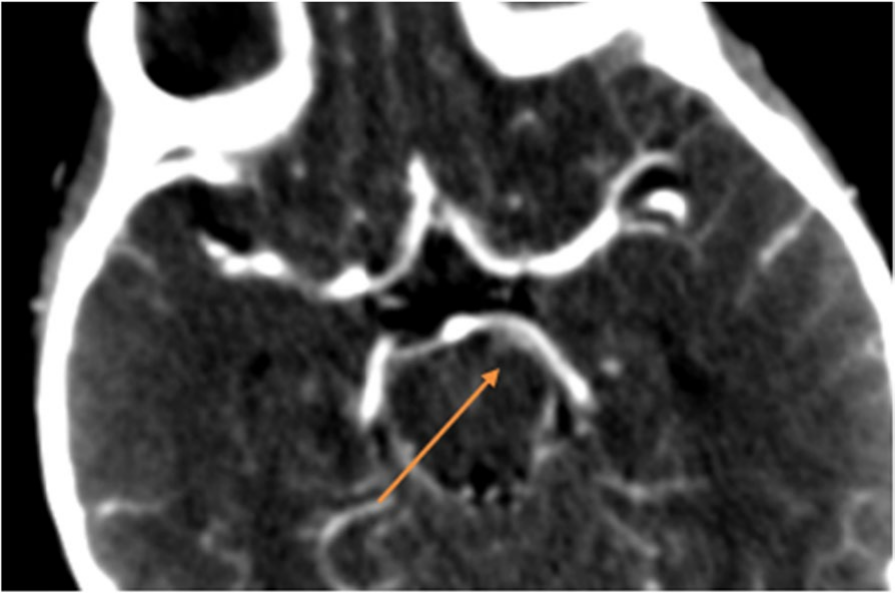
Minimal size irregularity of the P1 tract of the left posterior cerebral artery

**Fig. 3:**
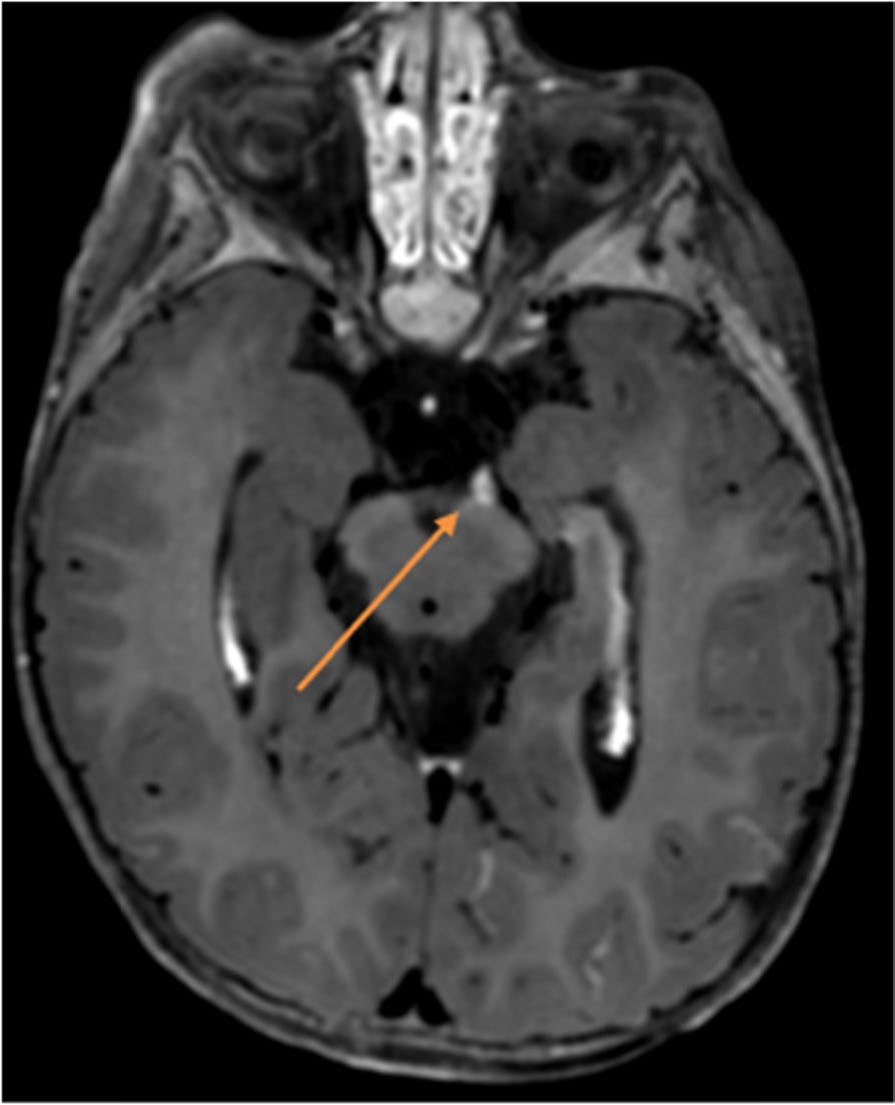
Sectorial slight thickening of the emergence of the left third cranial nerve, with a reduced post-contrast enhancement compared with the previous exam

Herein, we report the case of a 17-month-old male child referred to our Institute presenting gradual onset of mild eyelid ptosis and divergent strabismus of the left eye, preceded two days before by an episode of vomiting. A week prior to the hospitalization, an episode of inconsolable crying, lasting about two hours, occurred with loss of appetite during the following days. Neither fever nor other clinical findings were evident. The patient, third son, was born at term from Cesarean section after pregnancy complicated by placenta previa. Neonatal period was regular. Spherocytosis was diagnosed during the first months of life. His family history revealed spherocytosis (mother and sister) and Hashimoto’s thyroiditis (mother). At admission, physical examination was normal, except for eyelid ptosis and lateral deviation of the left eye due to mild medial rectus muscle deficiency and without pupillary dilation, suggesting the involvement of the third cranial nerve. Fundus examination was normal. C-reacting protein (CRP) was negative. Moreover, serological tests and autoimmune panel were negative. Brain magnetic resonance imaging (MRI), enhanced after contrast administration, and magnetic resonance angiography (MRA) were performed. They suggested a vascular anomaly, along the medial side of the left cerebral peduncle, referable to an arterial aneurysm nearby the ipsilateral third cranial nerve (Fig. 1). However, the angio-CT examination did not confirm the vascular anomaly, highlighting a minimal size irregularity of the P1 tract of the left posterior cerebral artery (Fig. 2). On the basis of MRI findings, a third cranial nerve neuropathy was suspected. About three weeks after hospital admission, left third oculomotor nerve ophthalmoplegia was no longer appreciable. One month later, a brain MRI was repeated and confirmed a sectorial slight thickening of the emergence of the left third cranial nerve, with a reduced post-contrast enhancement compared with the previous exam (Fig. 3).

**Fig. 4:**
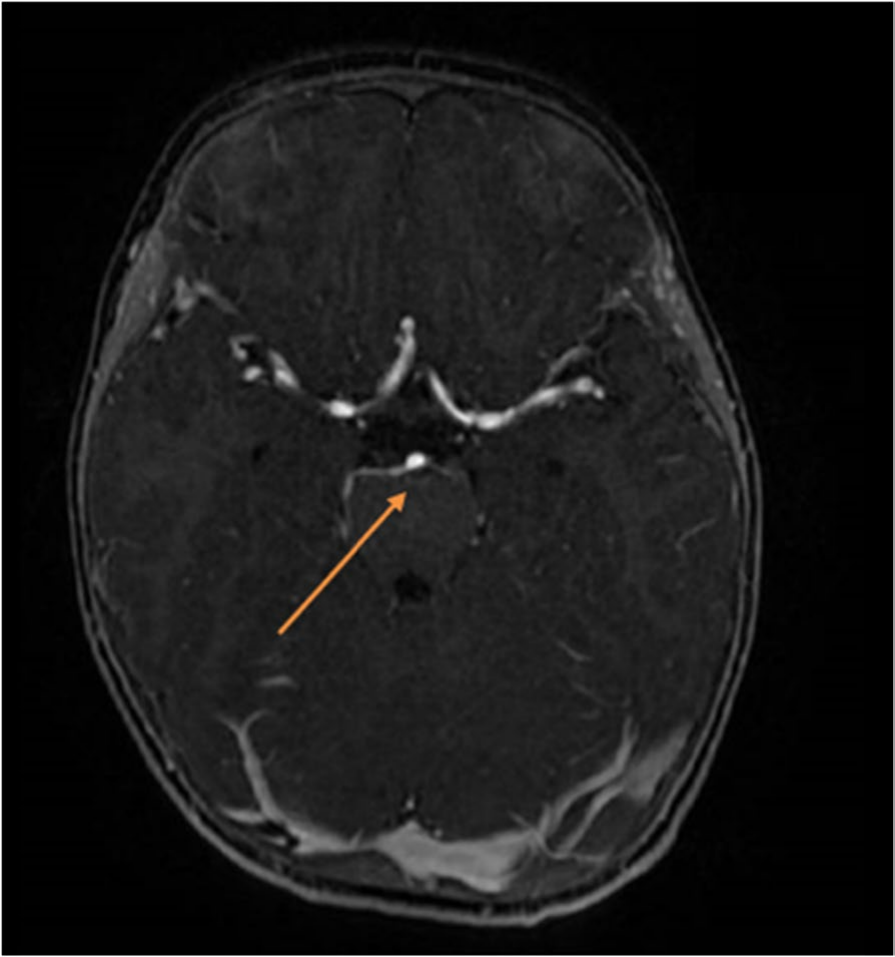
Complete resolution of the previous neuroradiological lesions

One year later, a brain MRI was repeated, showing a complete resolution of the previous neuroradiological lesions (Fig. 4). In relation to MRI findings and clinical situation, the reported case was highly suggestive of an episode of recurrent painful ophthalmoplegic neuropathy. Nonetheless, according to the diagnostic criteria proposed by the *International Classification of Headche Disorders* (ICHD) (2018) at least two attacks are necessary to confirm the diagnosis [19]. Other considerable, even highly improbable, hypothesis was a schwannoma of the third nerve. For a correct evaluation of the case, we planned a strict follow-up: after 18 months from the diagnosis, the patient had an episode of headache with inconsolable crying treated with paracetamol. During this episode, neurological examination was negative. No other similar episodes with ophtalmoplegia occurred and the neurological examination was negative. After 30 months, the child was conducted at our emergency department presenting again eyelid ptosis and divergent strabismus of the left eye, associated with vomiting and headache. During the hospitalization symptoms gradually resolved spontaneously with a total resolution. This second acute attack confirmed our already strongly suspected diagnosis of RPON.

## Discussion and conclusions

A diagnosis of RPON is always challenging, especially under 24 months of age. In fact, children are not able to describe headache and the first symptom is often irritability. Thus, in young children the diagnosis is also difficult to confirm unilateral headache as per International Classification of Headache Disorders (ICHD) criteria. It often happens that diagnosis is done in most of the cases in older age in follow up even though age of first presentation is before 2 years. Our case is highly suggestive of RPON but a definite diagnosis was not possible at the first attack [19].

**Fig. 5:**
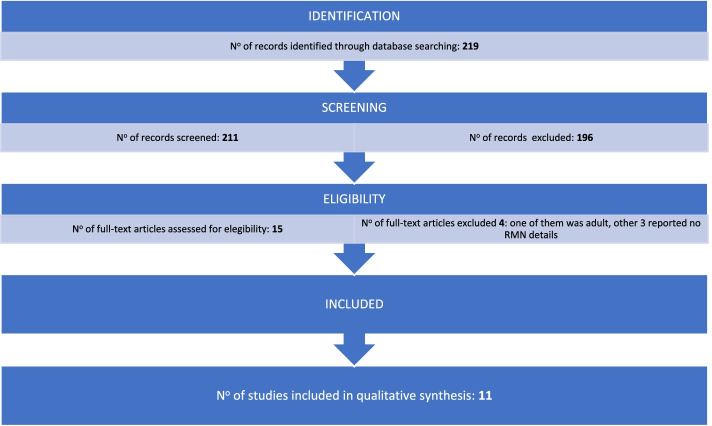
PRISMA (Preferred Reporting Items for Systematic Reviews and Meta-Analyses) statement

Third nerve thickening and post-contrast enhancement are suggestive of oculomotor nerve schwannoma, carcinomatosis, granulomatosis, inflammatory or infectious neuritis [20]. The case presented was from the beginning highly suggestive for RPON but MRI findings still have no relevance in the diagnostic criteria. Our case reported represents a diagnostic controversial: all symptoms and neuroradiological lesions were highly suggestive but no diagnostic possibilities were admitted during the first attack. In 1997 Wong and Wong [21] suggested to include these MRI findings associated to a single reversible episode of ophthalmoplegia as a supportive diagnostic criteria of RPON. Notwithstanding, neuroradiological imaging during the first episode have only suggested a probable and not a definitive diagnosis so far. It is important to highlight that in our case CT was positive, unlikely Ambrosetto et al. [2] reviewed, showing that CT is normally negative. We systemically reviewed in Pubmed all cases of RPON occurred within 24 months of age and we compared it with our patient. PubMed was searched for all cases of RPON using the search terms “ophthalmoplegic migraine OR recurrent painful ophthalmoplegic neuropathy”. Only articles in English or Spanish have been filtered. We performed the PRISMA (Preferred Reporting Items for Systematic Reviews and Meta-Analyses) statement (Fig. 5). Inclusion criteria were age (up to 24 months), presence of MRI findings and diagnosis of RPON, for these reasons, we excluded 208 records from database searching. Two reviewers independently agreed on selection of eligible studies and achieved consensus on which studies to include. The methodological quality of this systematic review has been assessed using the AMSTAR 2 [22] tool as a “low quality review”, since no randomized controlled trials (RCTs) are available to date on the scientific literature.

As Tables 1 and 2 show*,* we abstracted the following information: age at onset and current age; sex; cranial nerve (CN) involved (side); headache (side); associated symptoms: photophobia, phonophobia, nausea, vomiting, irritability, other findings; ocular findings: ocular symptoms/signs, diplopia, ophthalmoplegia, palpebral ptosis, pupillary dilation; MRI findings in the acute phase: nerve thickening, post-contrast enhancement; altered cerebrospinal fluid (CSF) if lumbar puncture performed; headache duration; ophthalmoplegia duration; interval between headache onset and ophthalmoplegia; time to resolution of symptoms/signs; therapy in the acute phase; follow-up; prophylactic therapy; control MRI, number of acute episodes; interval between episodes; comorbidity; family history of migraine. The median age at the first attack was 14,3 months. 69,2% of patients were females and 30,8% males. The cranial nerve involved was always the third one, except for two patients where it was not mentioned [13]. headache was the most frequent symptom, followed by nausea and vomiting. One third of patients presented associated symptoms such as photophobia, phonophobia and irritability. Ocular symptoms/signs were always present: ophthalmoplegia and palpebral ptosis were the most frequent ones, followed by diplopia. Unlikely reported by Huang [3], we found some patient who presented pupillary dilation. It is interesting to highlight the MRI findings: it had been showed a nerve thickening in 61,5% of cases and a post-contrast enhancement in 53,8% of patients. In cases where MRI was negative, it is important to understand whether the imaging was really negative or the timing was wrong. In fact, in some patients there is no evidence of MRI abnormalities neither during the interictal phase nor during the first attack and it could only be found after attacks [23]. Therapy in acute phase had been administered in 70% of patients using corticosteroids. In 50% of cases, at follow-up examination was noted a periodic recurrence of migraine with or without ophthalmoplegia. A limited number of patients (20%) had permanent neurological damage. Control MRI had been performed in 50% of cases. It showed in a limited number of patients (20%) normal findings and in the majority of them (80%) a persistent enlargement but to a lesser degree. Notably that family history of migraine was positive in 46,1% of patients and in most cases was on the mother side. We compared the characteristics of our patient with the ones of the review (MRI findings in the acute phase, symptoms and its duration and response to therapy). For the aforementioned reasons, we strongly supported from the first attack that this case was highly suggestive of RPON. We highlight that the first diagnostic hypothesis were aneurysm and schwannoma. Our work let us to extend the knowledge about RPON, suggesting to think at this diagnosis at its very first attack, even in presence of initial MRI findings referable to vascular anomaly or tumors as schwannoma. A relationship between RPON and schwannoma has been often discussed. In 2019 Petruzzelli et al. [24] reported a patient affected by RPON who developed, after years, a schwannoma of the third cranial nerve. They proposed two explanations of the aforementioned correlation. According to the first one, tumor could intermittently release chemical substances which stimulate trigeminal nerve receptors, leading to the headache. In this case, schwannoma would mimics RPON and it would be an initial manifestation of the tumor, which would be too small to be found in MRI. The second hypothesis, instead, considers RPON as an inflammatory cranial neuralgia and not a migraine. In this case, episodes of inflammation lead to demyelination and remyelination. Schwann cells proliferation could lead to the transformation into schwannoma. As a result, isolated oculomotor schwannoma could be considered as a long-term complication of RPON. Both hypothesis suggest the importance of serial brain MRIs in the long-term follow-up of RPON. In conclusion, our case, compared to the reviewed literature, a diagnosis of RPON was highly suggestive even at the first attack. Our work highlights the importance to consider RPON in presence of MRI findings and clinical symptoms referable to aneurysm or schwannoma. This review defines the characteristics of MRI findings at the first RPON attack occurred under 2 years of age. Although two attacks are necessary, it strongly suggests to consider RPON even at the first attack, in presence of described characteristics. Thus, as mentioned by Wong [21] and Yinglu [23], we suggest to add into the diagnostic criteria the MRI findings, including enhancement and thickening of the nerve involved. We analyzed the relationship between RPON and schwannoma. As proposed by Petruzzelli et al. [24], we are performing a long-term follow-up at our institute in order to prevent any complications.

## Data Availability

PubMed was searched for all cases of RPON using the search terms “ophthalmoplegic migraine OR recurrent painful ophthalmoplegic neuropathy”. Only articles in English or Spanish have been filtered.

## Abbreviations

RPON: Recurrent painful ophthalmoplegic neuropathy
OM: Ophthalmoplegic migraine
MRI: Magnetic resonance imaging
ICHD: International Classification of Headache Disorders
CN: Cranial nerve
CSF: Cerebrospinal fluid
CT: Computed tomography
